# Predicting the aesthetics of dynamic generative artwork based on statistical image features: A time-dependent model

**DOI:** 10.1371/journal.pone.0291647

**Published:** 2023-09-21

**Authors:** Pu Meng, Xin Meng, Rui Hu, Liqun Zhang

**Affiliations:** School of Design, Shanghai Jiao Tong University, Shanghai, China; Federal University of Paraiba, BRAZIL

## Abstract

Several automated aesthetic assessment models were developed to assist artists in producing artwork with high aesthetic appeal. However, most of them focused on static visual art, such as photographs and paintings, and evaluations of dynamic art received less attention. Dynamic visual art, especially computer-based art, includes diverse forms of artistic expression and can enhance an audience’s aesthetic experience. A model for evaluating dynamic visual art can provide valuable feedback and guidance for improving artistic skills and creativity, thereby benefiting audiences. In this study, we created eight generative artworks with dynamic art forms based on a commonly used method. We established a time-dependent model to predict the aesthetics based on visual features. We quantified the artworks according to selected image features and found that these features could effectively capture the characteristics of the changing visual forms during the generation process. To explore the effects of time-varying features on aesthetic appeal, we built a panel regression model and found that the aesthetic appeal of the generated artworks was significantly affected by their skewness of the luminance distribution, vertical symmetry, and mean hue value. Furthermore, our study demonstrated that the aesthetic appeal of dynamic generative artworks could be predicted by integrating image features into the temporal domain.

## Introduction

Visual art reflects human intelligence and imagination. Its visual nature allows people to evaluate it according to its external forms, leading to the formulation of formalism theory. Formalism suggests that the artistic value of a work of art depends on its formal aspects [1]. Formalists believe that visual art should be appreciated for its form, which can be experienced directly through sensations, typically sight or hearing [2]. Visual artworks are perceived not only through the arrangement or composition of their elements but also through sensual qualities such as rhythm and motion, especially for artworks involving dynamics. Film is a typical dynamic visual art form consisting of a series of still photographs. Formalist film theory evaluates how the nature and aesthetics of film contribute to its meaning by analyzing its style and narrative. The development of science and technology has broadened the methods for integrating dynamic features into visual art, leading to the production of various dynamic visual art forms.

Computer programs are common tools for producing dynamic visual art because they can create complex and interactive animations that might be difficult to produce with traditional methods. Generative art, which emerged in the 1960s, is a type of visual art that can be created programmatically [3]. It is defined as any art practice in which the artist uses a system, such as a set of natural language rules, a computer program, a machine, or another procedural invention, with some degree of autonomy to contribute to or produce a completed work of art [4]. The core mechanism for producing generative art is the generative system, which considers the initial states and entity behaviors [5]. By defining the initial states and entity behaviors, the entity states can be manipulated, and the generative system can update its outputs to create art. Based on the conceptualization of entities and their behaviors, generative art can be classified into two broad categories: artworks do or do not display self-organized patterns due to nonlinear interrelations among entities.

Generative art can be either static or dynamic. Differing from films, its dynamically generated form exhibits greater degrees of complexity and intricacy in its patterns and motions, especially in cases involving nonlinear interactions. Thus, new approaches are needed to evaluate its aesthetics. Since the visual outputs of most computer-based art generation methods are digital, some well-established image analysis methods may be useful for evaluating generative art. In the fields of computational and experimental aesthetics, several computational methods have been proposed to quantify images by various features to establish quantitative relationships between these features and digital image aesthetics [6]. These methods inherit the idea of formalism and establish more precise relationships between features and image aesthetics by converting visual forms into quantitative features. These features can be categorized into low-level features and high-level features. Low-level features, such as luminance, colors, edges, and textures, are directly extracted from the external forms of the input image and are broadly used for assessing the aesthetics of paintings [7–10] and photographs [11–15]. High-level features representing semantic information are used to examine how semantic information affects the aesthetics of photographs [16, 17] and paintings [18]. Attempts have been made to quantify the relationships between certain image features and personal aesthetic judgments of static generative art images [19, 20]. Effective methods for evaluating the aesthetics of dynamic generative art are still missing.

Neuroaesthetics research has demonstrated the impact of dynamic stimuli on people’s aesthetic perception. Previous studies have shown the differences in the aesthetic responses to images containing dynamic and static cues. For example, Di Dio et al. [21] found that aesthetic evaluations of portrait and landscape paintings involved the perception of motion information. Bara et al. [22] demonstrated that paintings with motion cues were preferred over those with static cues. Researchers have discovered the neural mechanisms underlying motion information processing in aesthetic evaluations using functional magnetic resonance imaging experiments. In addition to studying the dynamic information contained in static stimuli, neuroaesthetic researchers have investigated the aesthetic response mechanisms for dynamic stimuli. For example, Zhao et al. [23] explored the neural mechanisms underlying the aesthetic evaluation of dynamic landscapes, revealing that the perception mechanisms for static and dynamic landscapes are similar. Nevertheless, dynamic artistic stimuli induce more intense aesthetic responses due to the activation of brain regions associated with visual motion and emotion during the assessment of such stimuli. These findings suggest that dynamism plays an important role in dynamic image aesthetic evaluations.

Inspired by the quantitative study of the aesthetic appeal of photographs, several methods have been proposed to assess the aesthetic value of videos [24–26] by integrating their image features in the temporal domain. In the present study, since dynamic generative art could be regarded as a continuous sequence of images, we applied image analysis methods to dynamic generative art to explore how presenting image features in the temporal domain would reflect dynamic characteristics and affect aesthetics. We created eight generative artworks with a specific generative system and converted them into image sequences, which were rated by 1,701 participants through an online survey. Each artwork was quantified according to twenty-two image features. The experimental results show that the features effectively represent the visual variations during the generation process and can be used to predict the aesthetic evaluations of the artworks. These results facilitate the study of the relationships between aesthetic evaluations and the parameters of generative systems that produce generative art, allowing generative artists to enhance the aesthetics of their works by varying the parameters.

## Materials and methods

### Stimuli

**Fig 1:**
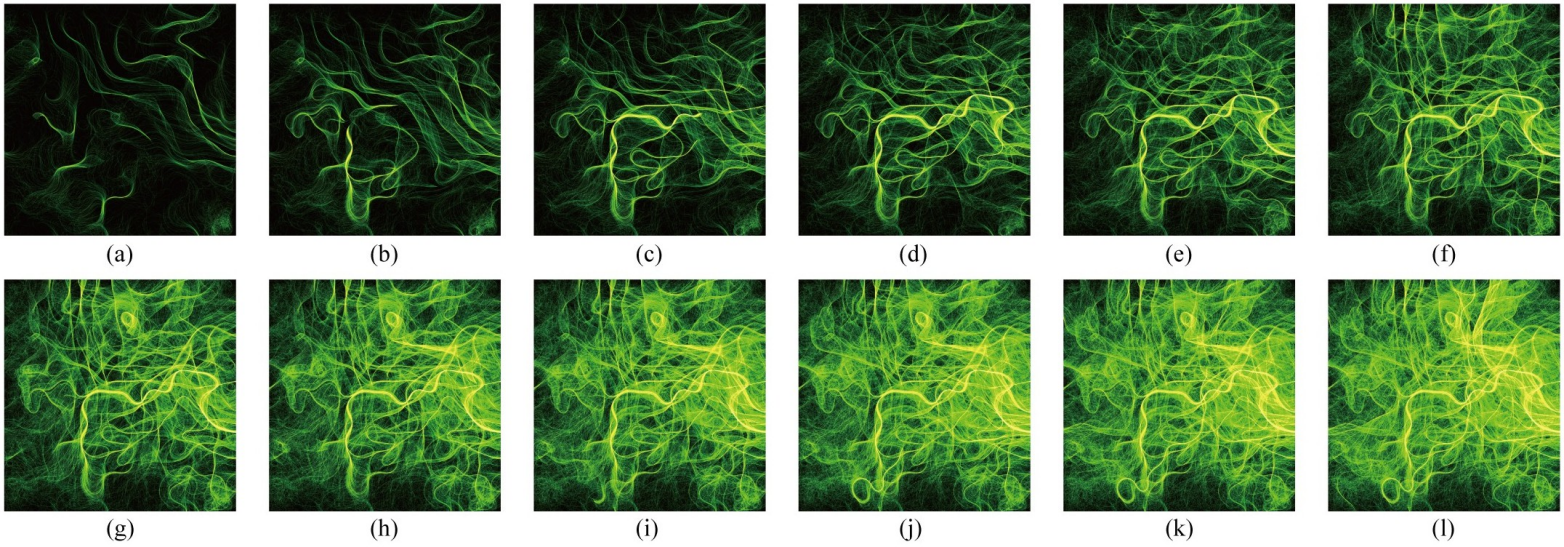
An image sequence of generative artwork. Panels (a)—(l) show the images sampled at 12, 24, 36, 48, 60, 72, 84, 96, 108, 120, 132, and 144 seconds, respectively.

Various generative systems can be used to create generative art. Since the present study cannot cover all systems, we employed the flow field (the Perlin noise field in this experiment), a widely used method in this domain, as the generative system to produce stimuli. The Perlin noise field is a flow field created using Perlin noise [27]; it is a stochastic sequence creator that can produce coherent and natural sequences of numbers. We introduced a two-dimensional vector space where all entities follow the direction of the underlying vector. The Perlin noise field controls the behavior of the entities, and its base structure causes the external forms of generative artwork to a self-organized pattern containing some implicit regularity, which is visually reflected by the entity motion throughout the generation process. To explore the relations between the external features and aesthetics of generative art, we created eight generative artworks and sampled them into image sequences as stimuli using Processing (version 3.5.4) [28]. One of these sequences is displayed in Fig 1, while the other sequences are presented in S1 Fig.

#### Generative art

Our art generation method includes two modules: generation and rendering modules. The generation module consists of a particle system and several parameters that control the particle behaviors. The rendering module includes a set of variables defining how to use the particles to create art. The particles are the entities in our generative art, whose states are characterized by their colors and positions. Their initial states are specified in the initialization phase and modified during the drawing phase by specific rules based on Perlin noise.

**Fig 2:**
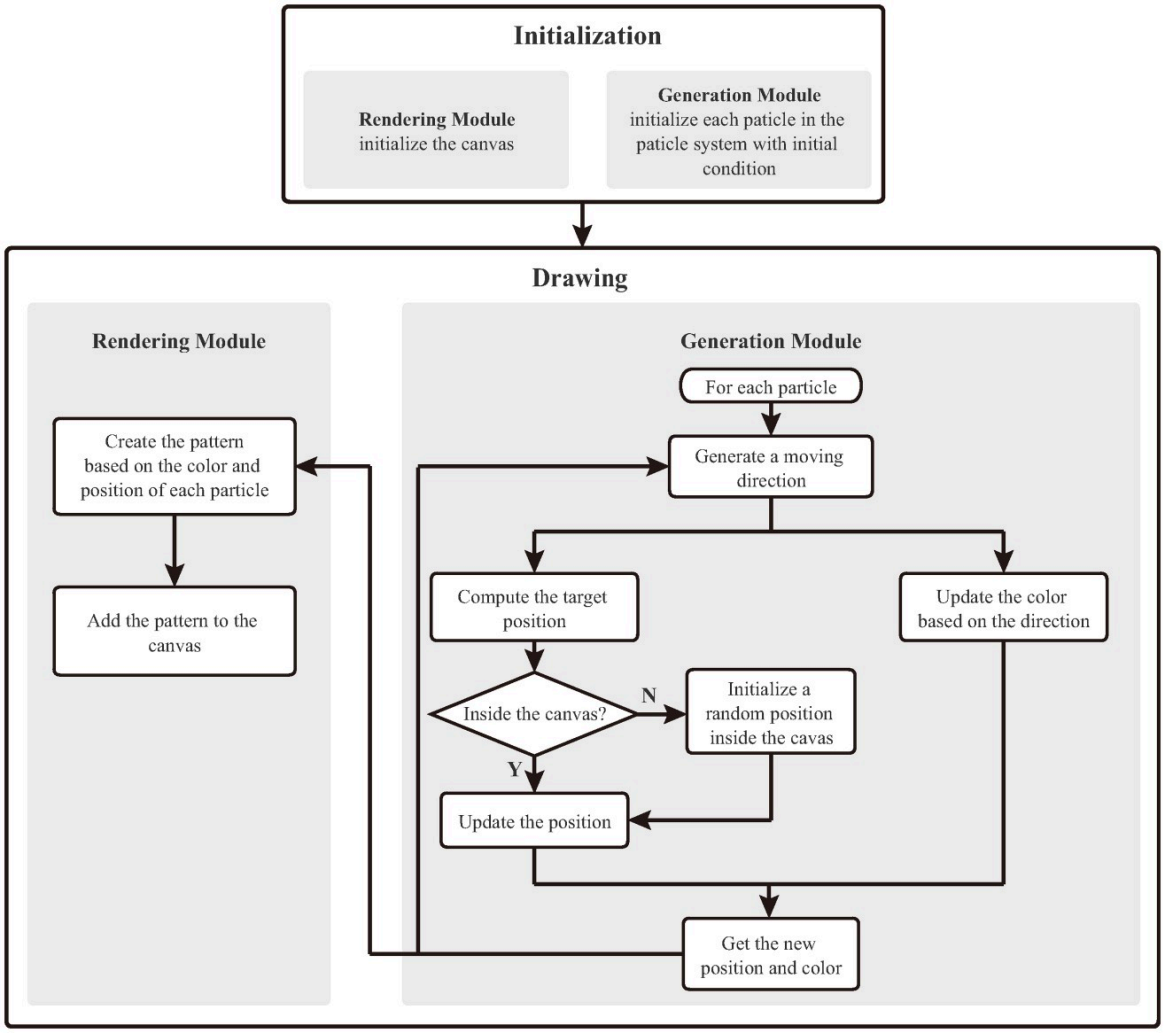
Overview of our art generation process. We first initialize the parameters of the particle system and the canvas and then continuously update the color and position of each particle and the canvas to create art.

Fig 2 presents an overview of our art generation method. In the initialization phase, the size (400×400 pixels in this experiment) and background color (black) of the two-dimensional canvas are specified. A blending mode is defined to calculate the pixel values in the canvas. In this mode, the final pixels (C) are computed by the source pixels (A) and the existing pixels (B) in the canvas, as shown in the following equation:

$$\begin{array}{cc} C_{channel} & =\text{min}(A_{channel}+B_{channel},255) \end{array}$$ (1)

where *C*_*channel*_ represents each channel of (C), i.e., its red, green, and blue channels, which are defined in the RGB color model. Each channel of (C) is independently determined by the sum of the corresponding channels of (A) and (B); the upper limit is 255, which represents white in the RGB model.

By sampling random numbers from a uniform distribution, *n* (*n* = 2,000) particles are assigned two-dimensional vector positions within the canvas. The chromatic attributes of each particle are defined by the hue, saturation, and brightness values. In practice, they are controlled by four parameters: saturation, brightness, and lower and upper limits of the hue range. In the drawing phase, the saturation and brightness parameters remain constant, while the hue parameter is changed within the specified range. Different initial conditions defined by the four color parameters lead to generative artworks with distinct color schemes.

**Fig 3:**
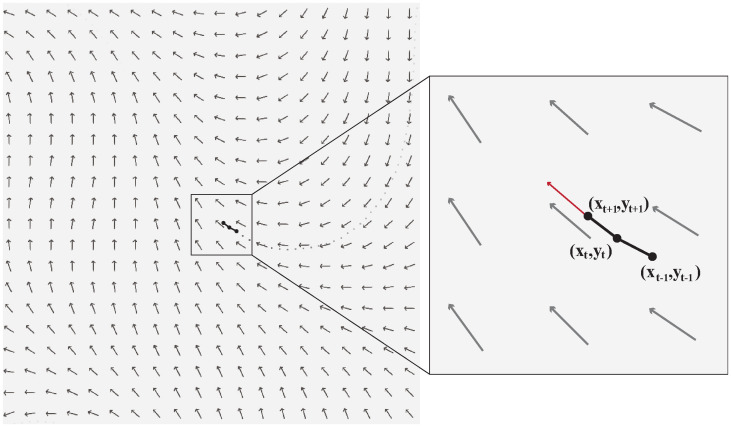
Perlin noise flow field. The gray arrows represent the directions of the force field. The movement of a particle in each frame is affected by the underlying force. The red arrow shows the movement direction of the particle in frame *t+1*.

In each frame during the drawing phase, the movement direction angle of each particle is computed using Perlin noise (see Fig 3) based on its position and the elapsed time of the phase. The target position coordinates are derived from the cosine and sine values of the angle. The position of a particle in frame *t+1* is determined using the following equations:

$$\begin{array}{cc} d_{t} & =perlin_{noise}\left(\frac{x_{t}}{width},\frac{y_{t}}{height},t\right)*c \end{array}$$ (2)

$$\begin{array}{cc} x_{t+1} & =x_{t}+\text{cos}\left(d_{t}\right)*v \end{array}$$ (3)

$$\begin{array}{cc} y_{t+1} & =y_{t}+\text{sin}\left(d_{t}\right)*v \end{array}$$ (4)

where *x*_*t*_ and *y*_*t*_ represent the x and y coordinates of the particle in frame *t*, respectively, *d*_*t*_ is the movement direction, which is restricted to the range of 0 to 90 degrees by the constant number *c*, and *v* is the velocity of the particle, which is set to 1 in this system. If the target position is outside the canvas, the particle initialization process will be repeated until a valid position is obtained.

The color variation of each particle is obtained by modifying its hue value according to the following equation:

$$\begin{array}{cc} hue_{i} & =\frac{perlin_{noise}\left(d_{t}\right)}{90}*\left(hue_{U}-hue_{L}\right)+hue_{L} \end{array}$$ (5)

where *hue*_*i*_ represents the hue value of particle *i* in frame *t*, and *hue*_*L*_ and *hue*_*U*_ are the specified lower and upper limits of the hue range, respectively.

For each particle, its movement is visualized by the rendering module, i.e. a circle is drawn on the canvas by its color with a diameter of one pixel at its location. The pattern drawn on the canvas can therefore represent the collective particle movement.

The drawing phase is configured to render 30 frames per second, allowing the generation and rendering modules to update their states 30 times per second, and therefore creating smoother and more fluent animations.

#### Image sequences

We employed a sampling approach to study the dynamic generative artworks by converting them into consecutive static images. To determine an appropriate sampling interval for noticeable perceptual differences between adjacent images within one sequence, a pre-experiment was conducted prior to the main experiment. Ten image sequences, that served as stimuli, were sampled from ten generative artworks, each with the same coloration, at intervals of 9, 12, 15, 18, 21, 24, 27, 30, 36, and 48 seconds, and contained ten static images.

**Fig 4:**
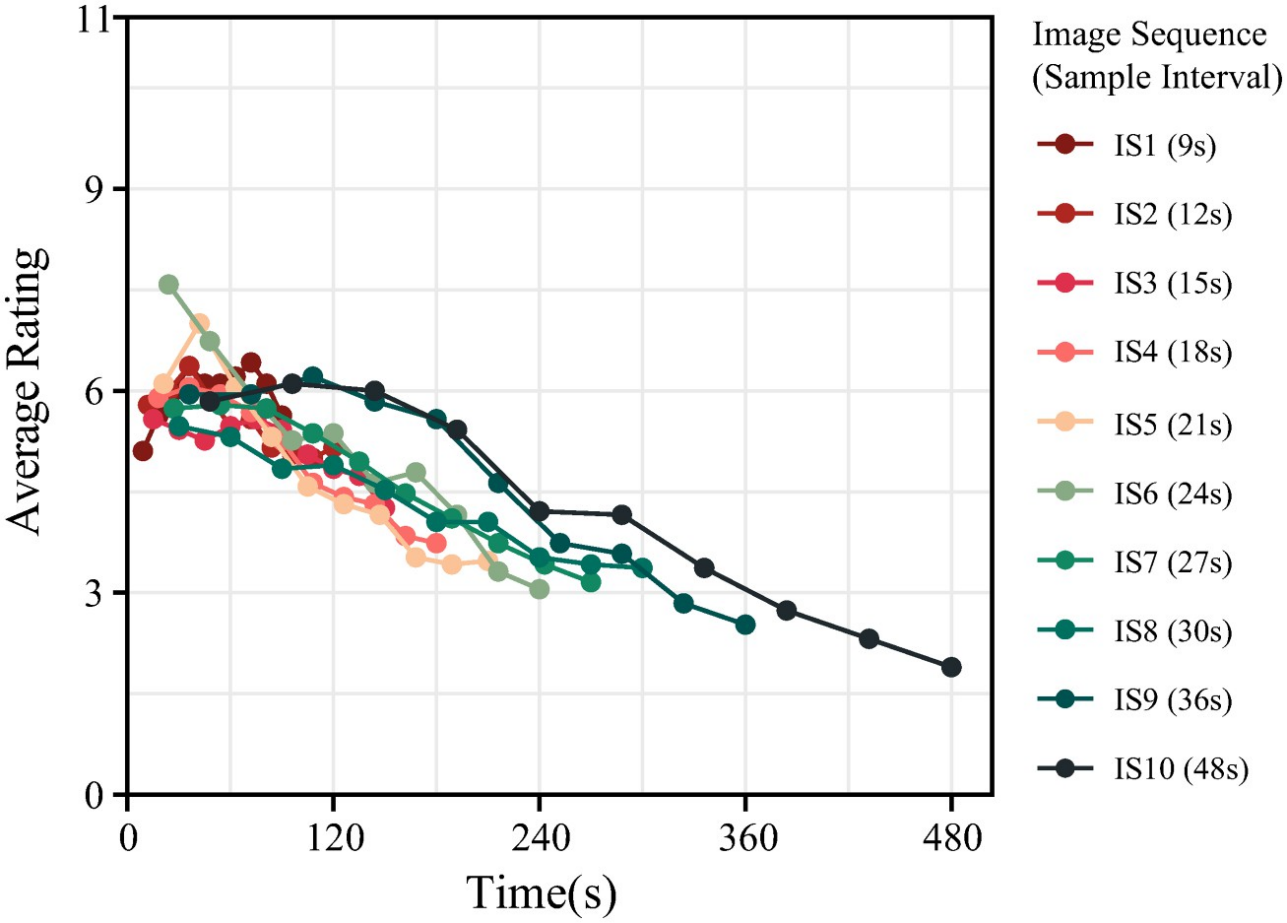
Aesthetic ratings of the image sequences over time. The different image sequences are represented by distinct colors, which correspond to different sampling intervals. The x-axis indicates the time point at which each image was sampled. The average rating of an image provided is represented by a point.

Nineteen participants rated each image based on its “visual aesthetic appeal” on a scale from 1 (ugly) to 11 (beautiful) through an online survey. The average ratings of the ten stimuli are presented in Fig 4. The ratings exhibit ascending and descending trends from 20 to 130 seconds, with the highest ratings occurring within the 24 to 60 seconds interval.

We would like to examine these trends with sufficient duration in the main experiment. We extracted twelve images from each artwork at 12-second intervals, resulting in a total duration of 144 seconds, which ensured perceptual differences between the adjacent images. To explore the potential influence of individual color preferences on aesthetic appeal, we created eight generative artworks with varying colors by specifying different color parameters in the initialization phase. We randomly assigned the saturation and brightness values (ranging from 0 to 100) and upper and lower limits (ranging from 0 to 360) of the hue within the specified ranges. This resulted in a total of 96 images with resolutions of 400 x 400 pixels, which were organized into eight distinct image sequences.

### Participants

An anonymous online survey(data collection began on March 18, 2023) was conducted to gather comprehensive data on the general evaluation of the stimuli by individuals. A total of 1,701 Chinese adults (54.1% female; age: M = 30.43, SD = 7.83) were recruited via the Internet irrespective of their vision status. They all fully completed the required tasks and were compensated for their involvement. Prior to the survey, all participants were informed of the study’s purpose, and they provided their informed written consent before participating. The studies involving human participants were reviewed and approved by the Institutional Review Board for Human Research Protections (IRB. HRP) of Shanghai Jiao Tong University.

**Fig 5:**
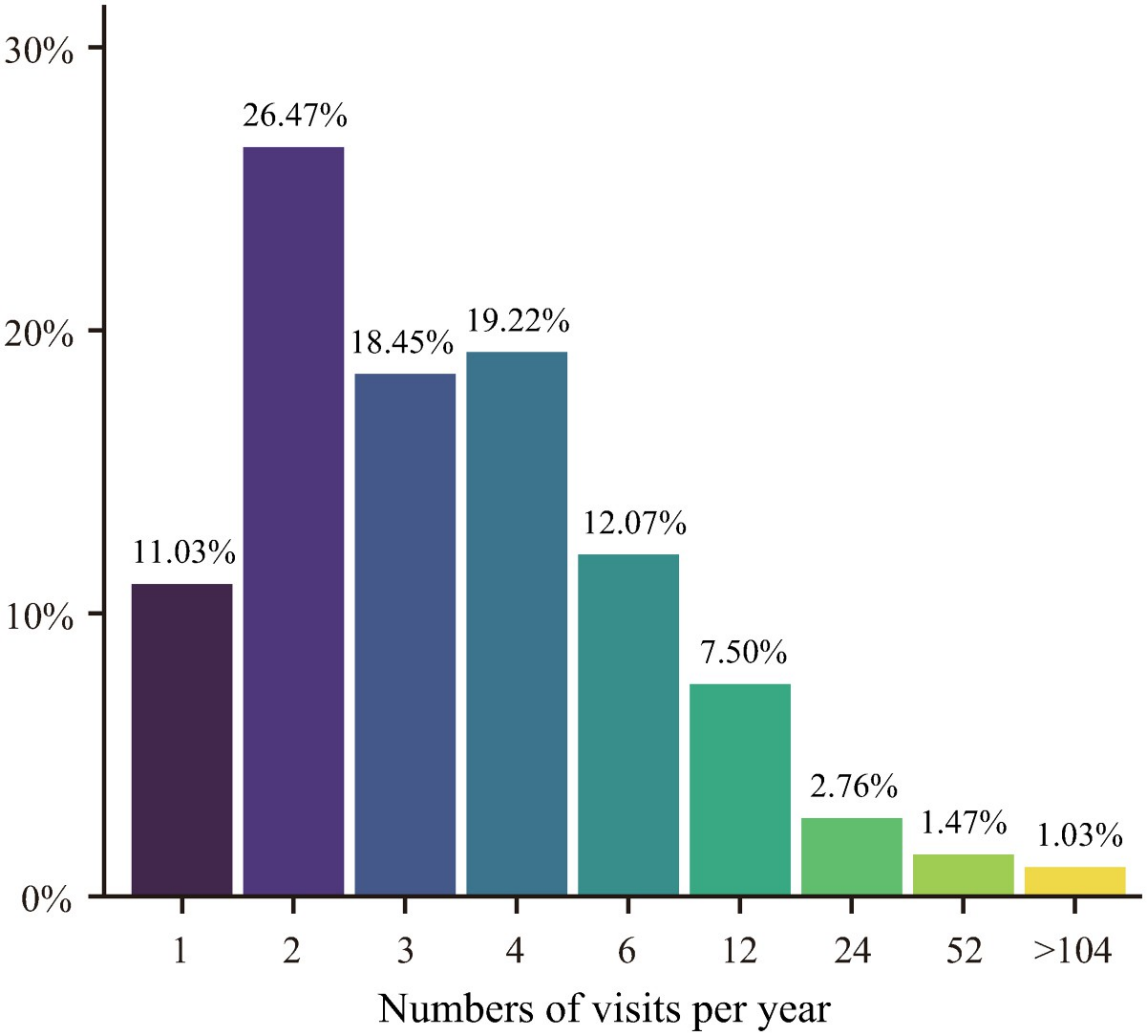
Frequency distribution of the number of art museum/gallery/exhibition visits reported by the participants.

Among the participants, 614 reported having professional training in art or design, and 476 reported having a career in art and design. Additionally, 161 participants reported no previous professional training in art or design. Among the participants, 1,160 reported visiting art galleries and exhibitions frequently and provided their numbers of visits to art museums/galleries/exhibitions per year, as depicted in Fig 5.

### Experimental procedure

At the beginning of the survey, the participants were randomly assigned to one of the eight image sequences and asked to read general instructions for the tasks. Then, the participants proceeded to the survey by clicking a button on the screen. The images in the sequence were displayed individually at a fixed position in the middle of the screen in chronological order so that the participants could notice the visual differences between adjacent images. The participants had at least 500 ms to form a general impression of each image before a button appeared to submit their rating and move to the next image. The participants rated each image based on its visual aesthetic appeal on a scale from 1 to 7 (1 = ugly, 4 = neutral, and 7 = beautiful). An average of 212 aesthetic ratings was obtained for each image sequence, providing a robust dataset for statistical analysis.

### Image features

#### Color

The preference for color is a key aspect of visual aesthetic appeal and art appreciation [29, 30]. However, they vary among individuals [31] and may influence aesthetic judgments of art images. To examine the effects of color on the aesthetic appeal of the stimuli, we used five features to measure the coloration in each image sequence.

**The number of colors** in an image is derived from the color parameters of the generative system, which are used to determine the color of each particle in the system. This metric was computed by counting the number of distinct colors that had been assigned to any particle during the generation process.

**Hue** is a key factor contributing to a pleasant aesthetic experience [31, 32]. We used the HSB color space to record colors and computed the mean and standard deviation of the hue value for each color used in the generative system. The mean value represents the holistic color impression of an image, while the standard deviation approximates the color harmony of the image. Based on Itten’s color harmony theory [33], which states that color harmony can be objectively measured by the relative positions of the hues on the color wheel, several harmonic templates [34] have been proposed to evaluate the color harmony of an image by comparing its color distribution with those of the templates. As a color set with a narrower hue distribution is more likely to match the templates, a hue distribution with a smaller standard deviation is associated with a more harmonious color combination.

**Saturation and brightness.** We also recorded the saturation and brightness values of the colors, as they are distinctive color features for the image sequences.

#### Luminance

Luminance is an important factor in characterizing the visual forms of an image. The aesthetics of paintings can be assessed by comparing the deviation in the luminance values of paintings with respect to natural scenes [35, 36]. Previous research on Western paintings has shown that more appealing artworks intentionally created by artists have different luminance statistics than natural scenes [37]. This difference reflects the perceptual properties of the human visual system. Hence, the luminance statistics of images are crucial for emulating human visual and aesthetic perception, and they are employed to evaluate the aesthetic appeal of paintings [7] and photographs [11, 12].

**RMS contrast.** We employed the root-mean-square (RMS) contrast of an image as a measure of its contrast dispersion. The RMS contrast, proposed by Peli [38], is the standard deviation of the grayscale pixel intensities. The grayscale images in the study were obtained by using the ITU-R 601–2 luma transform.

**Luminance distribution.** The luminance histogram, which represents the probability distribution of the pixel intensity, of each grayscale image is used to examine its intensity distribution. We determined two features according to the histogram: skewness and kurtosis. The skewness is used to determine the intensity of most pixels in the image, while the kurtosis of an image indicates the dispersion of its pixel intensity distribution. We calculated the skewness and kurtosis values of the grayscale images using the Cetinic and Grgic method [39].

#### Edge orientation

The distribution of the edge orientations across an image affects the image composition to some extent. Previous studies have found that natural scenes and Western paintings have low anisotropy [40], which means that their edges are evenly distributed in different directions. In this paper, two methods were employed to measure the edge orientation information in each image.

**Edge orientation distribution.** To quantify the uniformity of the orientations of the luminance edges in an image, we adopted the first-order entropy of the edge orientations (Redies et al. [41]) as a metric. This metric is derived from the Shannon entropy of the orientation histogram, which is obtained from the grayscale image edges detected by the oriented Gabor filters.

**Anisotropy** characterizes the statistical heterogeneity of the edges with different orientations in an image. Edges that are equally distributed in all directions have low anisotropy, while edges with certain directions have high anisotropy. To quantify the anisotropy of an image, we used the method proposed by Braun et al. [42] based on the pyramid histogram of the oriented gradients (PHOG) approach [43] to extract the histogram of oriented gradients (HOG) features and computed their standard deviations.

#### Self-similarity

People prefer images with statistical self-similarity [44], which stems from the adaptation of the human visual system to natural scenes due to evolution [45]. Thus, many scholars believe that images with the same statistical patterns as natural scenes are more appealing to humans [37]. Natural scenes are scale-invariant, meaning that their statistical properties do not change with the observation scale [46]. The PHOG method has been utilized to explore the statistical self-similarity of various kinds of images, including museum paintings [40], architecture images, and advertisements [42], by comparing the local and global parts of the image in terms of the HOG feature. We followed this method to determine the self-similarity of our generative artworks.

#### Complexity

Complexity is closely related to aesthetic perception; however, researchers have not yet reached a consensus on its relationship with aesthetics. Berlyne [47] suggested that people prefer stimuli that are neither too simple nor too complex, showing a reverse U-shaped curve between preference and complexity. This pattern can be observed in computer-generated images [48, 49]. However, other studies have found a linear relationship between preference and complexity, such as in Renaissance paintings [8] and advertisements [50]. Various methods can be used to measure complexity, such as image compression and fractal dimension algorithms [51]. In our study, we used two metrics to measure it.

**Edge density.** The perceptual complexity of an image can be estimated by analyzing its luminance gradients. Redies et al. [41] proposed a method that involves computing the edge density of an image by summing the responses to Gabor filters which could represent the responses of the human vision system.

**Complexity based on the gradient intensity.** In accordance with the method suggested by Braun et al. [42], the complexity of an image can also be determined by calculating the mean value of the magnitude of the pixels in the gradient image.

#### Symmetry

Human faces with symmetry are typically considered to be more attractive [52], which some researchers believe is because symmetry indicates good health [53]. Symmetry also influences the perceived beauty of abstract patterns [54] and art [9]. The symmetry of an image can be measured by comparing the pixels on both sides of a chosen axis that splits the image. In this study, we employed the method of Brachmann and Redies [55] and assessed image symmetry using a convolutional neural network (CNN) filter. The vertical symmetry, which indicates left-right mirror symmetry, was computed by comparing the filter responses of the original and mirrored images. We also calculated the horizontal and diagonal symmetry based on the filter outputs.

#### Saliency

Saliency estimation methods can detect salient regions, which are visually distinctive, based on local features such as color, texture, and shape [56]. These methods are useful for studying the aesthetic quality of photos and providing aesthetic evaluations [13] and classifications [14]. In our experiment, the stimuli had black backgrounds and light pixels that were visually distinctive; thus, the salient regions in the stimuli were mainly composed of light pixels. We used two saliency estimation methods, graph-based manifold ranking saliency (GBMR) and frequency-tuned (FT) methods, to extract saliency maps for each image. The GBMR method [57] is more accurate for single images, while the FT method [56] is better for maintaining interframe coherence in video sequences [58]. The saliency map is a grayscale image that shows the strength and size of the saliency according to the mean and standard deviation of the pixel values, respectively [14]. We used these two statistics as features to quantify the saliency.

#### Balance

The balance level of an image is an important factor that contributes to overall aesthetics in visual art [10] and photography [15]. Arnheim’s Gestalt theory of visual balance asserts that an image is considered balanced when its displayed landmarks are situated along any of the vertical, horizontal, or diagonal axes in the image. Jahanian et al. [15] used this theory to quantify the pictorial balance of a photograph by locating the image hotspots based on the saliency values. To measure the horizontal and vertical balance levels, we applied the method proposed by McManus et al. [59], which computes the center of mass (CoM) of an image by weighting each pixel’s intensity and its distances from the left and top edges of the image matrix. The CoM was then compared to the central axis value (CoM = 0.5) to calculate the deviation and balance scores for each axis.

### Data analysis

The data collection process was executed to ensure that there were no missing data in the dataset. Hence, all the data obtained in the experiment were included in the data analysis. We treated the sequence of each image as nominal data, and the attributes of each image as continuous data that comprise the sampling time, image features, and aesthetic ratings of each image. Statistical analyses were conducted to examine the associations among these attributes and the variations across different sequences.

The temporality of generative art implies that both the image features and ratings can be regarded as functions of time. These variables were derived from the image at specific sequences and temporal intervals, forming two dimensions. Hence, they can be treated as panel data. We developed a standardized panel regression model that estimated the aesthetics of the stimuli using the image features as explanatory variables. To improve the accuracy of the regression model, we employed two methods, regression subset selection [60] and principal component analysis (PCA), to select a subset of the predictors. Two criteria, the adjusted R^2^ ($R_{\text{adj}}^{2}$) and Akaike’s information criterion (AIC), were used to evaluate the model’s performance. A model with a higher adjusted R^2^ and lower AIC value is considered to perform better. The statistical analyses were performed using R [61] (version 4.1.3) with the Hmisc, lm, psych, leaps, and tseries packages.

## Results

### Image features

S1 Table presents the mean values and standard deviations of the image features produced for each image sequence (IS).

A nonparametric test is required because the Shapiro‒Wilk test indicates that the data deviates from normality. Also, eight groups corresponding to the eight ISs are examined in the study. Therefore, the Kruskal-Wallis test is applied to determine whether there are significant differences among the image features of the eight ISs. All features except for GBMR_std_ (H = 11.66, df = 7, *p* = 0.11) display significant differences between at least two ISs (*p* < 0.05). Notably, no significant differences are observed in the sizes of the salient regions obtained by utilizing the GBMR algorithm across different ISs. Furthermore, the color features, including the number of colors, hue_avg_, hue_std_, saturation, and brightness, significantly differ among all sequences (*p* < 0.001; pairwise Wilcoxon rank sum test with Bonferroni corrected), which aligns with the differences among the initial conditions.

The Spearman coefficient results obtained for the correlations among the image features are presented in S2 Table. The results strongly suggest that the image features cannot be regarded as independent variables.

Table 1 presents the correlations between time and the image features. The results indicate that except for color (number of colors, hue_avg_, hue_std_, saturation, and brightness) and balance (horizontal and vertical balance), all other features are significantly associated with time (*p* < 0.001). In particular, contrast and symmetry (symmetry_LR_, symmetry_UD_ and symmetry_LR_UD) exhibit strong positive correlations with time (r > 0.70), implying that contrast and symmetry increased as the stimuli evolved. In contrast, anisotropy is strongly negatively correlated with time (r = -0.73), with anisotropy decreasing with increasing time, which indicates a more uniform edge distribution across all orientations in the image. The luminance distributions (skewness and kurtosis) show moderate and inverse correlations with time (r = -0.67, r = -0.62). The complexity (edge density and complexity_GS_), first-order Shannon entropy, self-similarity, saliency strength (FT_mean_ and GBMR_mean_), and salient region size (FT_std_ and GBMR_std_) features exhibit moderate correlations with time (0.30 < r < 0.70). These findings demonstrate the significant correlations between time and the external forms of the stimuli captured by the image features. Therefore, artworks can be viewed as functions of time, which is consistent with the core mechanism of generative art, which produces evolving visual outputs.

**Table 1:** Spearman coefficients and corresponding 95% confidence intervals(CI) for the correlations between time and ratings according to the image features.

Image Feature	Time		Rating	
	r	95% CI	r	95% CI
Contrast	0.79***	(0.69, 0.87)	-0.72***	(-0.82, -0.60)
Skewness	-0.67***	(-0.78, -0.53)	0.70***	(0.56, 0.80)
Kurtosis	-0.62***	(-0.73, -0.46)	0.70***	(0.56, 0.80)
Horizontal balance	0.16	(-0.04, 0.35)	0.26*	(0.06, 0.44)
Vertical balance	0.06	(-0.15, 0.25)	0.11	(-0.09, 0.31)
First‒order entropy	0.45***	(0.26, 0.60)	-0.33**	(-0.50, -0.13)
Edge density	0.60***	(0.44, 0.72)	-0.74***	(-0.82, -0.61)
Symmetry_LR_	0.75***	(0.63, 0.83)	-0.73***	(-0.82, -0.60)
Symmetry_UD_	0.71***	(0.58, 0.81)	-0.68***	(-0.79, -0.55)
Symmetry_LRUD_	0.71***	(0.58, 0.81)	-0.71***	(-0.81, -0.58)
Self-similarity	0.69***	(0.56, 0.79)	-0.74***	(-0.83, -0.62)
Complexity_GS_	0.57***	(0.40, 0.70)	-0.73***	(-0.82, -0.60)
Anisotropy	-0.73***	(-0.82, -0.61)	0.78***	(0.66, 0.85)
FT_mean_	0.63***	(0.48, 0.75)	-0.75***	(-0.83, -0.63)
FT_std_	0.47***	(0.29, 0.62)	-0.64***	(-0.75, -0.49)
GBMR_mean_	0.63***	(0.48, 0.75)	-0.64***	(-0.75, -0.48)
GBMR_std_	0.35***	(0.15, 0.52)	-0.28**	(-0.46, -0.08)
Number of colors	0	(-0.20, 0.20)	0.17	(-0.03, 0.36)
Hue_avg_	0	(-0.20, 0.20)	0.40***	(0.21, 0.56)
Hue_std_	0	(-0.20, 0.20)	0.14	(-0.06, 0.34)
Saturation	0	(-0.20, 0.20)	0.09	(-0.12, 0.28)
Brightness	0	(-0.20, 0.20)	-0.15	(-0.34, 0.06)

Correlation significant at level

* p < 0.05,

** p < 0.01 and

*** p < 0.001.

### Aesthetic ratings

S3 Table presents the mean values and standard deviations of the average aesthetic ratings of the eight ISs for each image.

**Fig 6:**
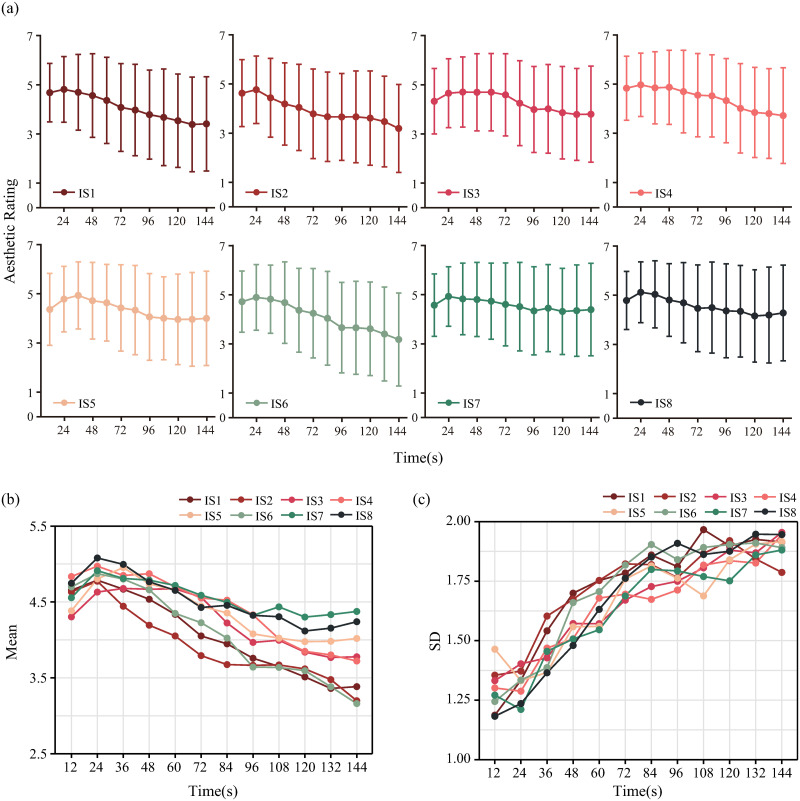
Aesthetic ratings of the image sequences over time. In panel (a), the average ratings of each IS are plotted as functions of time. The standard deviations are represented by the error bars. The trends over time are consistent with those in the pre-experiment. The mean ratings and the standard deviations for the eight ISs are plotted in panels (b) and (c), respectively.

The results demonstrate that the trend of the average aesthetic ratings over time is consistent with the conclusions drawn from the pre-experiment (refer to Fig 6). Specifically, as time increased, the aesthetic ratings initially increased, followed by a decrease, and the ratings peaked between 12 and 60 seconds. Notably, this trend is observed for all ISs, irrespective of the initial conditions. Additionally, the standard deviations of the aesthetic ratings increased with time, indicating a progressive amplification of the differences among the individual evaluations over time. These results suggest that as the stimuli dynamically evolved, the differences among the individual perceptions of the beauty of the present stimulus increased.

The average rating data exhibit non-normality, which necessitates the use of nonparametric tests. The pairwise Wilcoxon rank sum tests with Bonferroni corrected reveal no significant differences among the average aesthetic ratings of different sequences (*p* > 0.05). This finding indicates that the differences in the initial conditions of the generative system, namely, the colors, do not affect people’s aesthetic perceptions of the resulting artworks. In other words, individuals have consistent aesthetic judgments of the generative artworks produced by the same system. Thus, the aesthetic judgments in this study are not influenced by individual color preferences.

Furthermore, the aesthetic ratings exhibit a strong negative correlation with time (r = -0.81, *p* < 0.001). This result indicates that the individuals’ aesthetic evaluations of the stimuli gradually decreased over time.

### Image features and aesthetic ratings

The Spearman correlation coefficients for the associations between the image features and the aesthetic ratings are presented in Table 1.

The results suggest that the contrast, complexity (edge density and complexity_GS_), first-order Shannon entropy, symmetry (symmetry_LR_, symmetry_UD_ and symmetry_LRUD_), self-similarity, saliency strength (FT_mean_ and GBMR_mean_), and salient region size (FT_std_ and GBMR_std_) features are significantly negatively correlated with the aesthetic ratings (*p* < 0.01). In contrast, the luminance distribution (skewness and kurtosis), anisotropy, and hue_avg_ features are significantly positively correlated with ratings (*p* < 0.001). These findings indicate that images with lower contrast values, less complexity, less uniform edge orientation distributions, more asymmetry, less self-similarity, smaller salient regions, more balance between light and dark pixels, and yellow-green or bluish colors tend to elicit higher aesthetic ratings. Overall, these correlations reveal the importance of various image features that strongly affect the aesthetic appeal of generative artworks.

### Panel regression

**Fig 7:**
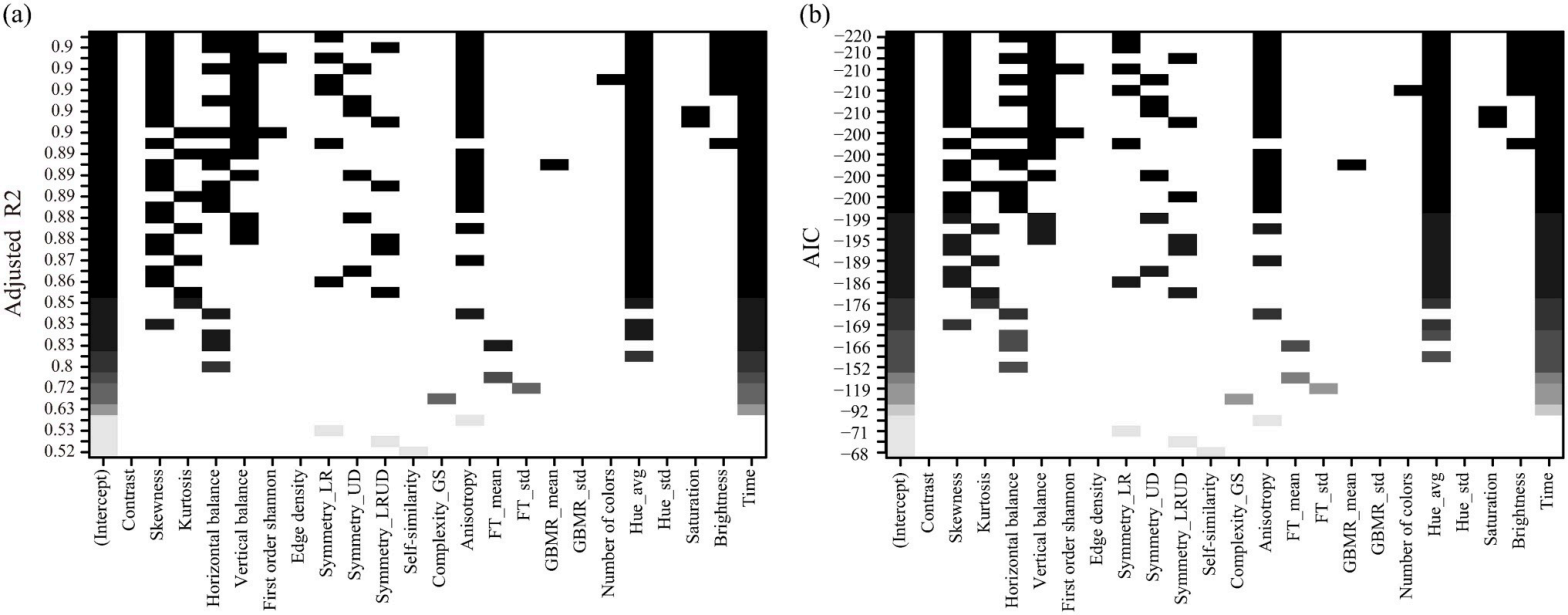
Regression subset selection results. Panels (a) and (b) show the AIC and adjusted R2 values for each regression model, respectively. The top row in each panel indicates the regression model that performed best.

In total, twenty-three variables, including twenty-two image features and time, were considered as predictors to establish a regression model. To address the multicollinearity among the predictors, regression subset selection was used to identify seven independent variables (see Fig 7) that were used for panel regression analysis: skewness, horizontal balance, vertical balance, symmetry_LR_, anisotropy, hue_avg_, and brightness. The variance inflation factor (VIF) values were assessed to ensure the absence of redundant predictors; all VIF values were below 5, indicating that the predictor subset was acceptable.

Three standardized regression models were constructed to examine the relationships between the variables. These models included a pooled regression model (adjusted R^2^ = 0.73, AIC= -118.84), an individual fixed-effects model (adjusted R^2^ = 0.86, AIC = -215.09), and an individual random-effects model (Nerlove’s transformation, adjusted R^2^ = 0.86, AIC = -207.28). Additional details about the regression models are presented in Table 2. The Hausman test results (chi-square = 3.12, *p* = 0.79) reveal that the individual random-effects model is the preferred choice. However, the assumptions of the random-effects model were not met: the differences across ISs should not influence predictor variables such as the brightness and hue_avg_.

**Table 2:** The adjusted R^2^ values and regression coefficients of the panel regression models according to the results of the regression subset selection.

Variable	Panel regression model		
	Pooled regression model	Individual fixed-effects model	Individual random-effects model (Nerlove’s transformation)
Adjusted R^2^	0.73***	**0.86** ***	0.87***
AIC	-118.84	**-215.09**	-207.28
(Intercept)	1.04E-14*		5.12E-11
Skewness	-2.13E-01	**-1.77E-01** *	-1.99E-01**
Horizontal balance	8.72E-02	**-5.94E-02**	-4.51E-02
Vertical balance	3.31E-01***	**5.05E-02**	6.83E-02
Symmetry_LR_	-5.43E-01***	**-1.04E+00** ***	-1.03E+00***
Anisotropy	4.96E-01***	**1.57E-01**	1.80E-01*
Hue_avg_	1.11E-01	**-1.76E+02** **	-1.17E+02**
Brightness	8.66E-02		-4.81E+01

Significant at level

* p < 0.05,

** p < 0.01 and

*** p < 0.001.

The model that was chosen for the final evaluation is indicated in bold font.

In addition to regression subset selection, we employed PCA to determine the most effective predictors. The first five principal components with a cumulative proportion of 0.86 are chosen as predictors, as indicated by the loading matrix presented in S4 Table.

Based on the five principal components, we developed three regression models: a pooled regression model (adjusted R^2^ = 0.69, AIC = -106.61), an individual fixed-effects model (adjusted R^2^ = 0.78, AIC = -171.07), and an individual random-effects model (Nerlove’s transformation, adjusted R^2^ = 0.79, AIC = -163.99). Additional information about the regression models is presented in S5 Table.

As the fixed-effects model based on regression subset selection outperforms the model based on PCA (adjusted R^2^: 0.86 > 0.79, AIC: -207.28 < -163.99), the former is employed as the regression model. Additional information about the models is presented in Table 3. The normality assumption of the residuals is not violated according to the Shapiro-Wilk test (W = 0.99, *p* = 0.31). Grubbs’s test does not identify any outliers in the residuals (G = 2.90, *p* = 0.15).

**Table 3:** Fixed-effects panel regression model.

Variable	Coefficient	SE	95% CI	Adjusted R^2^	F-test
Skewness	-1.77E-01*	7.62E-02	(-3.26E-01, -2.73E-02)	0.86	F_(6,82)_=99.90***
Horizontal balance	-5.94E-02	7.89E-02	(-2.14E-01, 9.53E-02)		
Vertical balance	5.05E-02	7.30E-02	(-9.25E-02, 1.94E-01)		
Symmetry_LR_	-1.04E+00***	8.82E-02	(-1.21E+00, -8.64E-01)		
Anisotropy	1.57E-01	9.06E-02	(-2.08E-02, 3.34E-01)		
Hue_avg_	-1.76E+02**	5.58E+01	(-2.85E+02, -6.62E+01)		
	Median	IQR	RSS	RSE	
Residuals	6.08E-03	4.03E-01	9.01E+00	3.08E-01	

Significant at level

* p < 0.05,

** p < 0.01 and

*** p < 0.001.

SE, standard error; CI, confidence interval; IQR, interquartile range; RSS, residual sum of squares; RSE, residual standard error.

Six variables, namely, skewness, horizontal balance, vertical balance, symmetry_LR_, anisotropy, and hue_avg_, are selected as predictors to estimate the aesthetic ratings of the stimuli. The regression model results reveal that skewness (b = -0.18, t = -2.32, *p* = 0.02), horizontal balance (b = -0.06, t = -0.75, *p* = 0.45), symmetry_LR_ (b = -1.04, t = -11.76, *p* < 0.001), and hue_avg_ (b = -175.56, t = -3.15, *p* = 0.002) are negatively correlated with the aesthetic ratings. This suggests that images with lower absolute skewness values, horizontal CoMs closer to the left sides of the images, less vertical symmetry, and more yellow-green and bluish colors are more aesthetically pleasing. In contrast, vertical balance (b = 0.05, t = 0.69, *p* = 0.49) and anisotropy (b = 0.16, t = 1.73, *p* = 0.09) are positively correlated with the aesthetic ratings. This indicates that images with vertical CoMs closer to the bottoms of the images and specific orientation distributions tend to receive higher aesthetic ratings.

## Discussion

The present study built a model for evaluating the aesthetics of generative artworks based on their quantitative image features. Our model is particularly to the created stimuli and may not apply to other generative art types due to their inherent diversity. Future research is needed to establish a general evaluation model that can be applied to various generative artworks.

### Utilizing image features to predict the aesthetic ratings of generative art

The findings of our study suggest that the aesthetic ratings of our generative artworks are consistent despite their varying initial conditions. We argue that dynamic processes, which are the core of generative art [5], are crucial in the aesthetic perception of generative art. Therefore, we propose that the appreciation of generative artworks is based on the understanding of these dynamic mechanisms. Hence, we incorporated time to assess the aesthetics of generative art and considered both the aesthetic ratings and image features of generative art as functions of time. We built a panel regression model and identified six independent variables that could predict the aesthetics of generative art: skewness, horizontal balance, vertical balance, symmetry_LR_, anisotropy, and hue_avg_.

The results suggest that generative artworks with lower skewness values tend to receive higher aesthetic ratings. A lower absolute skewness value implies that the image has an approximately normal pixel intensity distribution. Among our stimuli, decreased skewness corresponds to an increase in the number of light pixels and a nearly balanced ratio between the light and dark pixels. Previous research [37] has shown that artworks from different cultures and periods tend to have low-skewness luminance histograms, which suggests that low-skewness images may be preferred by artists and viewers. This may be because these images can be processed by the visual cortex more efficiently than high-skewness images [62]. The gradual decrease in the skewness of our stimuli over time suggests a balance between light and dark pixels in the images. These images are effectively processed by the visual system, contributing to the preference for images with similar characteristics. Overall, our findings suggest that individuals tend to prefer low-skewness images.

According to Arnheim’s Gestalt theory of visual balance, an image with its CoM aligned with Arnheim’s horizontal and vertical axes tends to receive higher aesthetic ratings. However, in the present study, balance features do not significantly affect the aesthetic ratings of the stimuli. This may be because the CoM does not change linearly over time, and its deviations from the axes are small. Therefore, balance features may not significantly influence the aesthetic ratings of generative art in this particular case.

Vertical symmetry is inversely correlated with aesthetic ratings, suggesting a preference for vertical asymmetry in generative art. This phenomenon may be explained by the dual process perspective of fluency theory, as proposed by Graf and Landwehr [63]. According to this theory, asymmetry is thought to activate controlled processing, leading to an increased appreciation for images with low processing fluency. Furthermore, the preference for asymmetrical abstract art is influenced by individual art expertise, as demonstrated by Mayer and Landwehr [64], who showed that individuals with lower levels of art expertise tended to prefer more symmetric images. However, we noted that our study did not account for the influence of individual differences on the aesthetic evaluation results. Therefore, future studies may benefit from including participant-related variables to investigate the interactions between individuals and image properties and how they influence aesthetic perception.

In the present study, there is no statistically significant evidence for the correlation between anisotropy and aesthetic evaluations at the 5% level. Our generative artworks do not contain visually identifiable semantic content. In accordance with prior research [65] on aesthetic judgments and abstract paintings without semantic content or identifiable objects, no correlations between anisotropy and aesthetic ratings are identified for formal abstract artworks. Further research is needed to confirm the effect of anisotropy on the aesthetic assessment of generative art. Hue_avg_, which measures the holistic color impressions of stimuli, is significantly correlated with the aesthetic ratings of the stimuli. By limiting the hue range of each artwork to yellow, green, blue, and red, we find that a smaller mean hue value corresponds to a higher aesthetic appreciation. Specifically, we observe a preference for yellow-green colors, contrasting with previous findings suggesting that blue is the most preferred color and that yellow-green is the least preferred color [66]. Additionally, Mallon et al. [65] suggested a strong preference for yellowish abstract artworks. These discrepancies may be due to the specific stimuli used in the present study. Overall, our results suggest that generative artworks with yellow-green colors are associated with higher aesthetic ratings.

### Dynamic changes in the image features in generative art

To measure how the visual forms of our generative artwork evolve, we applied image analysis methods to extract features that capture the shapes, colors, and compositions of the image and examined their temporal patterns. The results reveal that these features can effectively represent the visual variations in generative artworks.

Most image features, except for color and balance features, exhibit significant relationships with time. This lack of correlation between time and color features can be attributed to the limitations of our generative system; because the available colors are iterated through and drawn on the canvas during the first time interval, the color features of the output images remain constant for the subsequent 12 to 144 seconds. Therefore, it is not surprising that no significant correlations between the color features and time.

Balance features measure the degrees to which the CoM deviates from the left column and top row of the image matrix in the horizontal and vertical directions, respectively. Balance features are expected to be positively correlated with time; i.e., the CoM should approach Arnheim’s horizontal and vertical axes. However, the results do not indicate a monotonic relationship between the CoM position and time. Instead, the CoM (horizontal: M = 5.03E-01, SD = 3.71E-03; vertical: M = 5.00E-01, SD = 3.82E-03) fluctuates in a small range around both axes throughout the 144-second duration, indicating that the CoM is determined in the early stage and remains essentially stable over time. This CoM consistency may be attributed to the design of the generative system, as the particle movements are driven by random numbers produced by Perlin noise and thus exhibit a certain regularity. This regularity appears as consistency in the stimuli, which may be captured by the balance features.

Besides the implicit consistency, the explicit variations caused by particle movements are visually presented by the changes in the intensity and composition of the pixels. At the intensity level, these changes are reflected as increases in the number of light pixels. They affect the statistics of the luminance distribution in the image, as indicated by the correlation between time and luminance features. They also lead to the enlargement of the strength and size of the saliency map, which is reflected by the positive correlations between saliency features and time. This can be understood from the fact that light pixels in the stimulus, visually prominent relative to the black background, become more visible during the process. At the composition level, some global changes occur in the stimuli, and they can be visually perceived as the addition of more lines to the canvas during the generation process. In the early stages of the generation process, the visual output contains only a limited number of edges, which are predominantly oriented in specific directions. However, as time progresses, the number of edges in the image increases, and these edges become more randomly distributed, resulting in a more uniform edge orientation distribution. This visual change is reflected by variations in the edge orientation features and complexity features over time. Additionally, the correlations between time and the symmetry and self-similarity features demonstrate that the visual output becomes more symmetrical and self-similar over time.

One major limitation of the present study pertains to the inadequate representation of the dynamic nature of generative art through the ISs. In the online survey utilized to collect IS aesthetic ratings, participants were instructed to rate the same generative artworks, which were presented as twelve images in sequence and 144-second GIF images, on a 7-point scale. The statistical analysis reveals significant differences between the average ratings of the IS and the GIF image for each artwork, indicating the influence of the presented forms on the aesthetic perception of generative art. Thus, evaluating a static IS is unlikely to capture individual aesthetic judgments when observing continuously changing artwork fully. Moreover, behavioral reactions may not accurately convey the specific responses of individuals to aesthetic presentations. Therefore, to more precisely examine the aesthetic perception of generative art, other perceptual data, such as physiological reactions, should be incorporated into the experimental design.

## Conclusion

In this paper, we presented a novel approach to evaluate the aesthetics of dynamic generative artwork produced by autonomous systems. We hypothesized that the aesthetics of generative art were influenced by the dynamic processes underlying its creation, and these processes could be understood by the image features extracted from the artworks evolving over time. To test this hypothesis, we evaluated both the aesthetic ratings and the image features of generative artworks as functions of time and used a panel linear regression model to examine their relationships. The aesthetics of generative art showed less dependence on the initial conditions but more on the dynamic mechanism of the generative system. We concluded that the temporal dimension was essential for appreciating generative art and our method could provide insights into the aesthetic principles of such systems.

However, due to the constraints imposed by the stimuli used in this study, the research findings may apply to only the studied generative systems. The applicability of the findings to other generative art remains an open question. Future research will focus on examining the generalizability of the model for evaluating the aesthetics of generative artworks beyond the specific stimuli used in this study. By analyzing the relationships between the evaluation model and generative system variables, we aim to develop a generative system that generates artworks that are more aesthetically pleasing to individuals. Work along this line will contribute to the development of a general aesthetic evaluation model for generative art.

## Supporting information

S1 Fig Image sequences (ISs) of the generative artworks. Panels (A)-(G) show IS 2, 3, 4, 5, 6, 7, and 8, respectively.
(DOCX)

S1 Table Mean values and standard deviations of the image features for each IS. (XLSX)

S2 Table Spearman coefficients of the correlations among the twenty-two image features. (XLSX)

S3 Table Mean aesthetic ratings and standard deviations for the images in each IS. (XLSX)

S4 Table The loading matrix of the principal component analysis. Each element represents: how much each original variable contributed to the corresponding principal component.
(XLSX)

S5 Table The adjusted R^2^ values and regression coefficients of the principal component regression models. (XLSX)

S6 Table Average aesthetic ratings and the image features calculated for each image in the ISs. (XLSX)

S7 Table Individual aesthetic ratings for each image in the ISs. (XLSX)
