# Supplementary material for: Predicting the aesthetics of dynamic generative artwork based on statistical image features: A time-dependent model
Source: PLoS One. 2023 Sep 21;18(9):e0291647. doi: 10.1371/journal.pone.0291647 (PMC10513343; doi:10.1371/journal.pone.0291647)

**Supplementary Figures**

**Figure S1.** The image sequences (IS) of the generative arts. Panels (A) - (G) show the IS 2, 3, 4, 5, 6, 7, and 8 respectively.


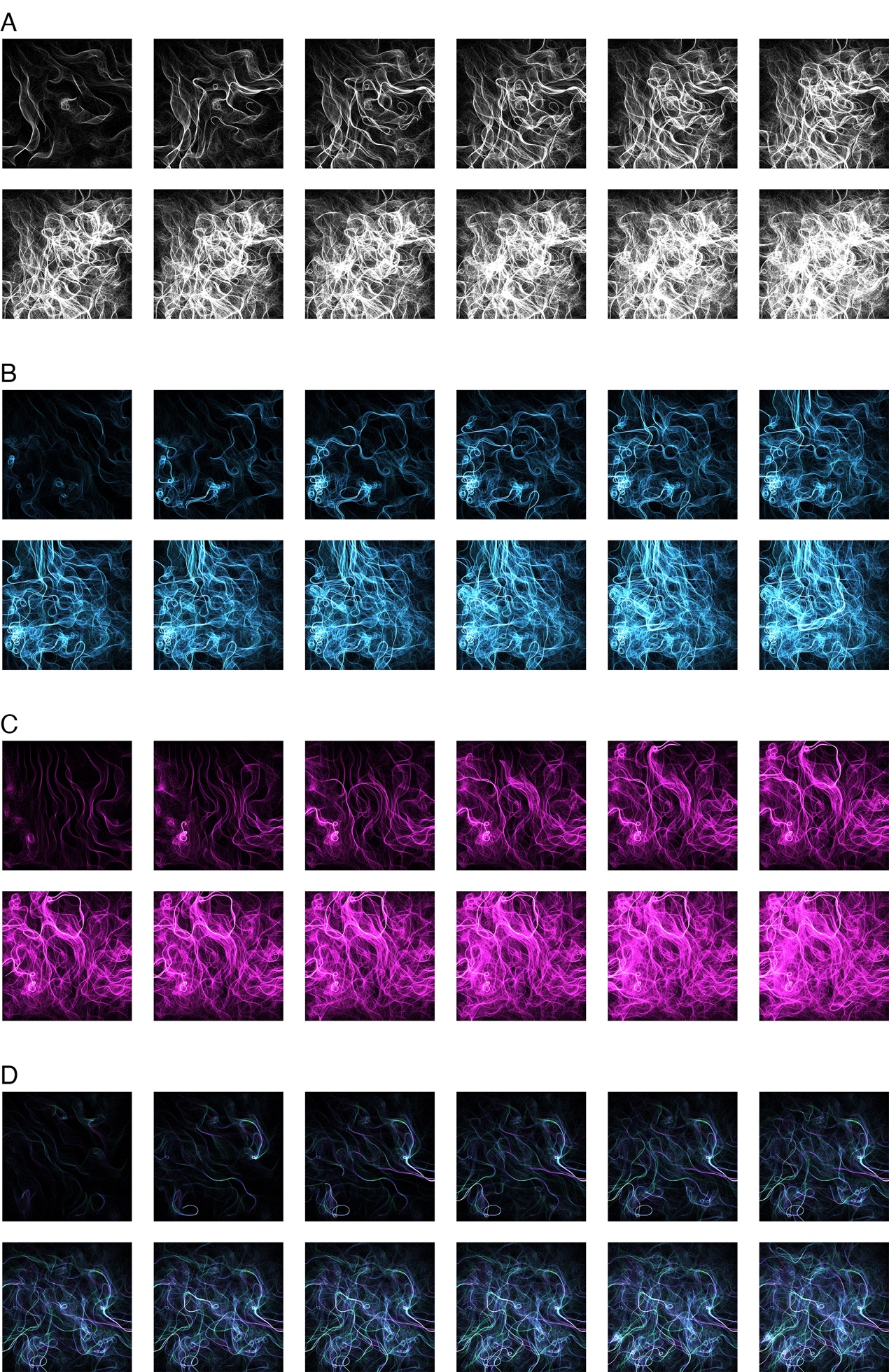

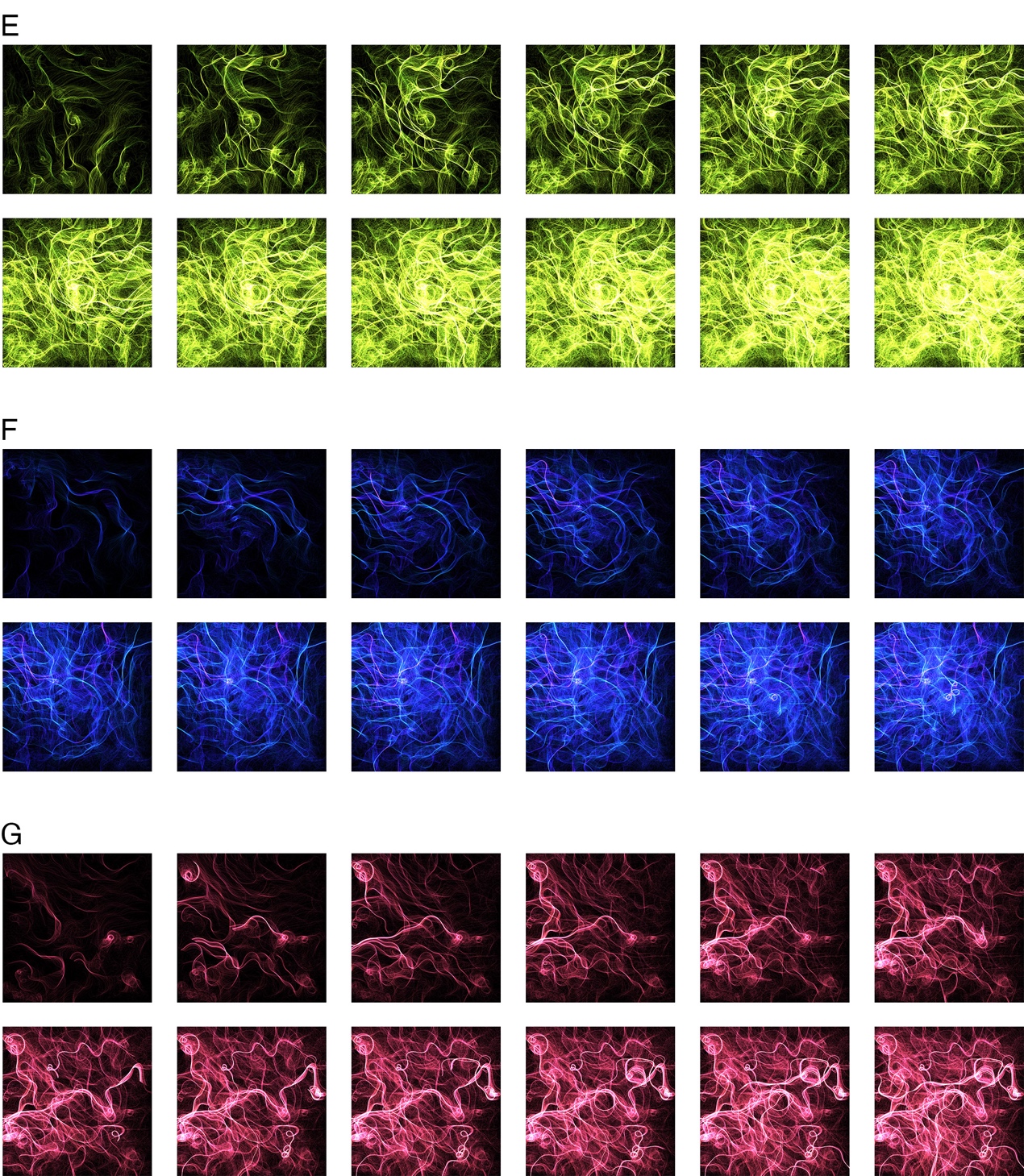

Supplement: S1 Fig — Panels (A)-(G) show IS 2, 3, 4, 5, 6, 7, and 8, respectively. (DOCX) [file pone.0291647.s001.docx]
